# Clinical Effect of Traditional Chinese Medicine Shenhuang Granule in Critically Ill Patients with COVID-19: A Single-Centered, Retrospective, Observational Study

**DOI:** 10.4014/jmb.2009.09029

**Published:** 2021-02-09

**Authors:** Jun Feng, Bangjiang Fang, Daixing Zhou, Junshuai Wang, Dengxiu Zou, Gang Yu, Yikuan Fen, Dan Peng, Jifa Hu, Daqian Zhan

**Affiliations:** 1 Department of Critical Care Medicine, Tongji Hospital, Tongji Medical College, Huazhong University of Science and Technology, Wuhan 430030, P.R. China; 2 Department of Emergency Medicine, LongHua Hospital, Shanghai University of Traditional Chinese Medicine, Shanghai 200032, P.R. China; 3 Office of Academic Research, Tongji Hospital, Tongji Medical College, Huazhong University of Science and Technology, Wuhan 430030, P.R. China

**Keywords:** Coronavirus disease 2019, traditional Chinese medicine, Shenhuang Granule, inflammation

## Abstract

The coronavirus disease 2019 (COVID-19) pandemic has become a public health emergency of global concern. In China, traditional Chinese medicine has been widely administered to COVID-19 patients without sufficient evidence. To evaluate the efficacy of Shenhuang Granule (SHG) for treating critically ill patients with COVID-19, we included in this study 118 patients who were admitted to the ICU of Tongji Hospital between January 28, 2020 and March 28, 2020. Among these patients, 33 (27.9%) received standard care plus SHG (treatment group) and 85 (72.1%) received standard care alone (control group). Enrolled patients had a median (IQR) age of 68 (57-75) years, and most (79 [67.1%]) were men. At end point of this study, 83 (70.3%) had died in ICU, 29 (24.5%) had been discharged from ICU, and 6 patients (5.2%) were still in ICU. Compared with control group, mortality was significantly lower in treatment group (45.4% vs. 80%, *p* < .001). Patients in treatment group were less likely to develop acute respiratory distress syndrome (ARDS) (12 [36.3%] vs. 54 [63.5%], *p* = 0.012) and cardiac injury (5 [15.1%] vs. 32 [37.6%], *p* = 0.026), and less likely to receive mechanical ventilation (22 [66.7%] vs. 72 [84.7%], *p* = 0.028) than those in control group. The median time from ICU admission to discharge was shorter in treatment group (32 [20–73] days vs. 76 [63–79] days, *p* = 0.0074). These findings suggest that SHG treatment as a complementary therapy might be effective for critically ill adults with COVID-19 and warrant further clinical trials.

## Introduction

Severe acute respiratory syndrome coronavirus-2 (SARS-CoV-2) is the novel coronavirus first detected in Wuhan, China, that causes coronavirus disease 2019 (COVID-19) [1]. Since initial detection of the virus, more than 26,760,000 cases and 876,000 deaths have been reported worldwide as of September 2020 (World Health Organization, COVID-19 outbreak situation 2020, accessed 7 September 2020). Unfortunately, the number of cases and deaths continues to rise every day. So far, the COVID-19 pandemic remains a global challenge.

Manifestations of SARS-CoV-2 infection are broad and variable, from asymptomatic carriers to pneumonia and fulminant disease, including acute respiratory distress syndrome (ARDS), sepsis, and ultimately, multisystem organ failure with many patients being admitted to intensive care unit (ICU) [2, 3]. At present however, there are no specific therapeutic drugs available for COVID-19. Currently, supportive therapies including oxygen support are the cornerstone for management of COVID-19 [4, 5]. In addition, antiviral drugs, immune modulators, and convalescent plasma are under investigation [6-9]. In China, greater than 85% of SARS-CoV-2-infected patients have received Traditional Chinese Medicine (TCM) treatment [10]. However, reports evaluating the efficacy of TCM herbal formula in treating patients hospitalized with COVID-19, especially in critically ill patients, are limited.

TCM has been used effectively to combat epidemic infectious diseases for thousands of years. To date, hundreds of herbal TCM formulae have been developed to prevent and treat epidemic infections. Based on “Truncation and Reversion” strategy and the theoretical understanding of the herbal properties within TCM, we developed the Shenhuang Granule (SHG) [11]. As described in our previous paper, SHG is composed of *Panax ginseng*, *Rheum palmatum* L. stem, *Sargentodoxa cuneate*, *Taraxacum mongolicum*, *Aconiti Lateralis Radix Praeparata*, and *Whitmania pigra* Whitman [12]. *Panax ginseng* and *Rheum palmatum* L. stem have been found to show antiviral activity [13-15]. Jinhong Decoction, composed of *Rheum palmatum* L. stem, *Sargentodoxa cuneata*, and *Taraxacum mongolicum*, could inhibit inflammatory cytokines such as TNF-α, IL-6, and IL-8 and protect against excessive inflammatory response in severe infectious diseases [16, 17]. *Aconiti Lateralis Radix Praeparata* shows a significant effect in rheumatoid arthritis by inhibiting the concentrations of IL-1β, TNF-α, and IFN-γ [18]. *Whitmania pigra* Whitman has been widely employed in decoction for the treatment of blood stasis syndrome for many years in China and its enzyme extracts suppress LPS-induced upregulation of inflammatory factors in rat vascular smooth muscle cells [19]. These pre-clinical and clinical studies indicated that some herbs of SHG could directly kill bacteria and inhibit viruses while other herbs could regulate inflammation response and maintain a balance between pro-inflammatory and anti-inflammatory mediators, with no serious adverse reactions observed [13-19]. Therefore, SHG treatment can be expected to have several beneficial effects on clinical outcome in patients with severe COVID-19. In this report, we evaluate the efficacy of combined SHG and standard care for treating critically ill adults with COVID-19.

## Methods

### Study Design and Participants

This single-center, retrospective, observational study was done at Tongji Hospital (Wuhan, China), which has two, large ICUs and was designated to treat patients with severe COVID-19. We recruited all of the adult (aged ≥18 years) inpatients admitted to ICU from January 28 to March 28, 2020, who were diagnosed with laboratory-confirmed COVID-19, according to World Health Organization interim guidance. All patients received standard care according to the National Guideline of Diagnosis and Treatment of COVID-19 (Trial Version 7). The standard care included timely provision of effective oxygen therapy, circulatory support, renal failure and renal replacement therapy, blood purification treatment, immunotherapy and other therapeutic measures such as short-term use of glucocorticoids. The patients recruited from one of the ICUs at Tongji Hospital received two sachets of SHG per day, from ICU admission to death or discharge, together with standard care. Patients with severe primary disease including malignant tumor, blood diseases, severe liver disease and known allergies to one or more substances in SHG, as well as women during pregnancy or lactation, were excluded from SHG treatment. We compared outcomes in patients who received standard care plus SHG (treatment group) with those in patients who received standard care alone (control group). Patients who either died within 48 h after being admitted to ICU, or those who received other herbs and participated in other clinical trials, or those admitted without key information, were excluded from the analysis. Follow-up continued through April 22, 2020. The study was approved by the Ethics Committee of Tongji Hospital(HSZY-PJ-2020-001-01). Informed consent was obtained from each patient or the patient’s legally authorized representative.

### Study Medications

The SHG comprises 50 g of *Panax ginseng* C. A. Mey, 40 g of *Rheum palmatum* L. stem, 30 g of *Sargentodoxa cuneata* stem, 30 g of *Taraxacum mongolicum*, 50 g of *Aconiti Lateralis Radix Praeparata* and 6 g of *Whitmania pigra* Whitman, which are packaged into two sachets. (The detailed formula and medicinal product certificate were provided in a separate document for reviewing purposes). SHG was manufactured based on the Pharmacopedia of the People’s Republic of China and provided by Beijing Tcmages Pharmaceutical Co., Ltd. The granule was dissolved in warm water and taken orally by patients. For those critical patients with difficulty taking medication, the granule solution was administered through feed tube.

### Data Collection

Data was extracted independently by two researchers using a standardized form. The data obtained included demographic characteristics, chronic medical histories, vital signs at ICU admission, laboratory finding, chest radiographs or computed tomography (CT) scan, treatment, and complications as well as outcomes of each patient. Laboratory tests for IL-6, IL-10, and TNF-a were not available for all patients.

### Outcomes

The primary outcome was mortality after ICU admission. Secondary outcomes were incidence of organ dysfunction/failure including ARDS, acute kidney injury, acute hepatic injury, acute cardiac injury, coagulopathy and shock, the proportion of patients requiring mechanical ventilation, and length of ICU stay. ARDS was defined according to the Berlin definition [20]. Acute kidney injury was diagnosed according to the KDIGO Clinical Practice Guideline [21]. Acute liver injury was defined as ALT and/or AST ≥ 2 times the upper limit of normal, with total bilirubin ≥ 2 times the upper limit of normal and/or international normalized ratio ≥ 1.7. Acute cardiac injury was defined by troponin elevation above the 99th percentile. Coagulopathy was defined as prothrombin time (PT) and/or activated partial thromboplastin time (APTT) more than 1.5 times the normal control.

### Statistical Analysis

Continuous variables were expressed in mean ± SD or median (IQR), as appropriate. Categorical variables were presented as number (percentage). Comparisons between continuous variables used Student‘s *t*-test or Mann-Whitney test. Comparisons for categorical variables were performed by using the chi-squared test or Fisher exact test. Kaplan-Meier method was used for survival data and the log-rank test was used to compare the survival times between both groups. A two-sided *p*-value of less than 0.05 was considered statistically significant. Statistical analyses were performed using Graphpad Prism, version 8.0.

## Results

### Baseline Characteristics 

**Fig. 1:**
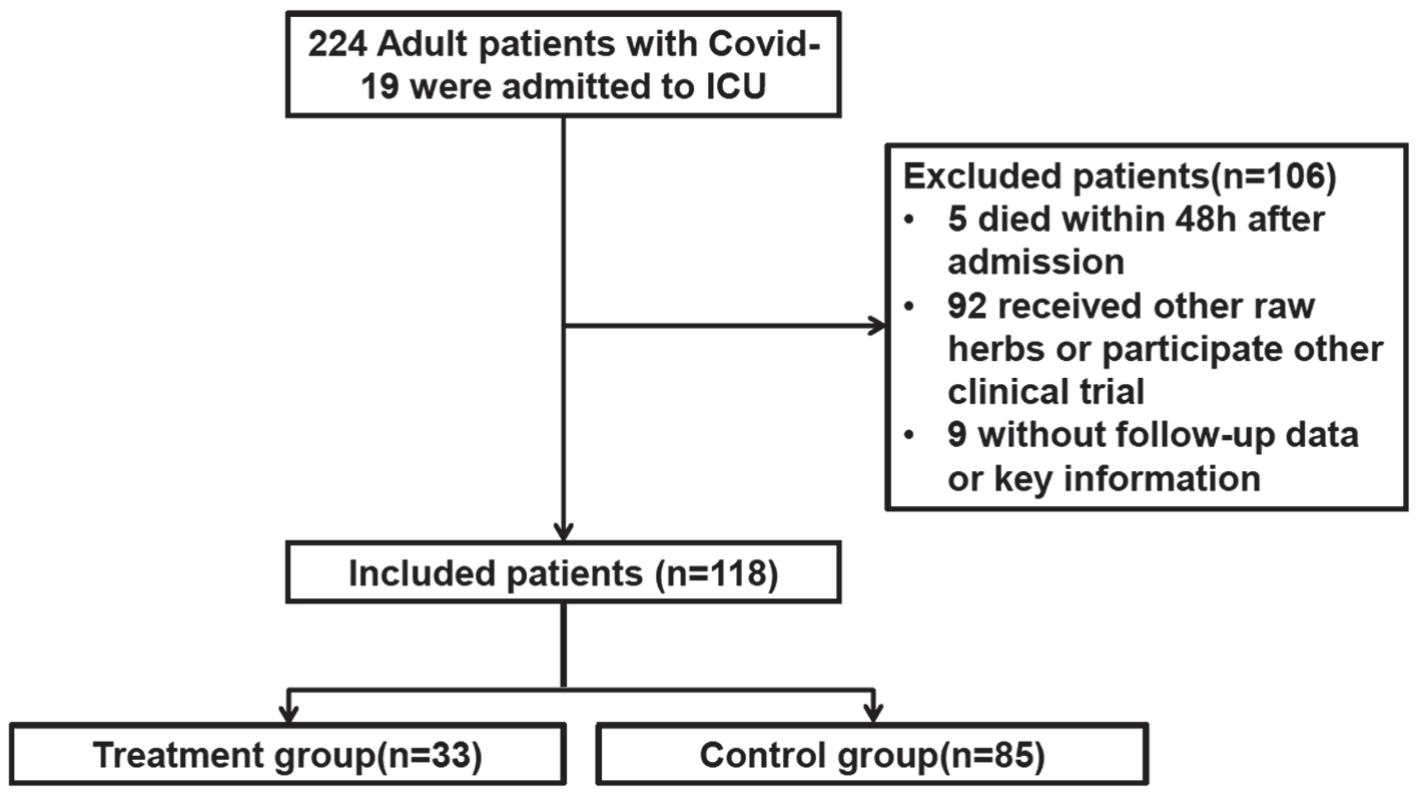
CONSORT flow diagram.

From January 28, 2020 to March 28, 2020, 224 patients with COVID-19 were admitted to ICU of Tongji Hospital. Of these patients, a total of 106 patients were excluded from this study. Five patients died within 48 h after being admitted to the ICU; 92 patients received other raw herbs or participated in other clinical trials. For 9 patients, follow-up data or key information were not available. Thus, 118 patients were included in the analysis (Fig. 1).

Of the118 patients, 33 (27.9%) received standard care plus SHG and 85 (72.1%) received standard care alone. The median duration of administration of SHG was 9 days (IQR, 6-14 days). Overall and per-group baseline characteristics are presented in Table 1. Baseline characteristics show an overall median age of 68 years (IQR, 57-75 years) and a predominance of men (79 [67.1%]). Hypertension (52 of 118 [44.0%]), diabetes (25 of 118 [21.1%]), and coronary heart disease (21 of 51 [17.7%]) were the most frequent comorbidities. The demographics, clinical characteristics, laboratory findings and concomitant treatment were comparable in both groups. (*p* > 0.05, Table 1).

### Outcomes

**Fig. 2:**
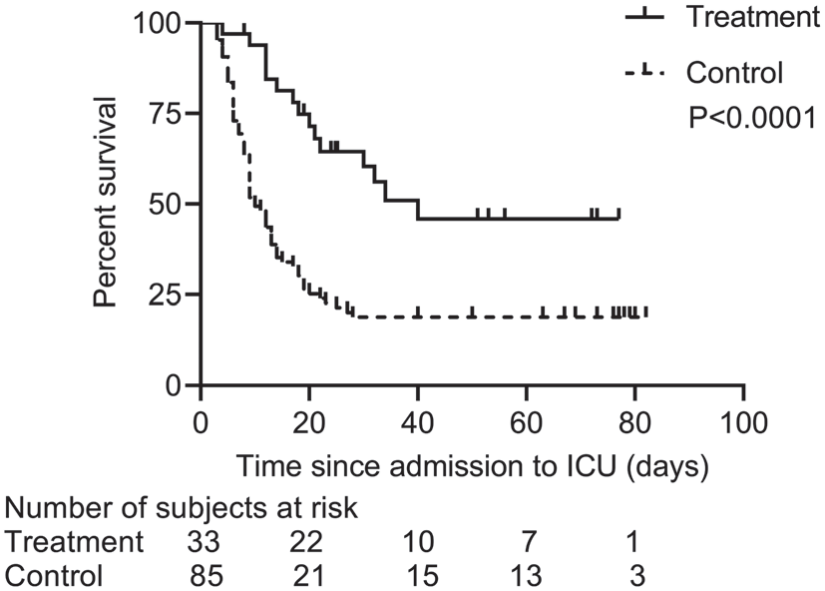
Kaplan–Meier curves of survival rate according to the treatment (log rank test *p* < 0.0001).

As of April 22, 2020, among the 118 patients, 83 (70.3%) had died in the ICU, 29 (24.5%) had been discharged from the ICU, and 6 patients (5.2%) were still in the ICU, with a median follow-up time of 13.5 days (Table 2). ICU mortality in treatment group was significantly lower compared with control group (45.4% vs 80%; *p* < .001). Moreover, among patients who died in the ICU (*n* = 83), the median survival time was longer in treatment group than that in control group (18 [12-30] days vs. 9 [6-13] days; *p* < 0.001); while among patients discharged from the ICU, the median length of stay in ICU was significantly shorter in treatment group as compared with control group (32 [20-73] days vs. 76 [63-79] days, *p* = 0.0074) (Table 2). Kaplan-Meier analysis showed that the survivor probability of patients with COVID-19 in treatment group was significantly higher than that of patients in control group (*p* < 0.001) (Fig. 2).

Of the 118 critically ill patients, about half of patients had organ dysfunction, including 66 (55.8%) with ARDS, 37 (31.3%) with cardiac injury, 36 (30.5%) with acute kidney injury, 29 (24.5%) with coagulopathy, 27 (22.8%) with shock, and 18 (14.2%) with liver dysfunction. Compared with control group, treatment group patients were less likely to develop ARDS (12 [36.3%] vs 54 [63.5%], *p* = 0.012) and cardiac injury (5 [15.1%] vs 32 [37.6%], *p* = 0.026). The occurrence rate of other complications was similar in both groups (*p* > 0.05). Twenty (16.9%) patients were treated with high-flow nasal cannula oxygen therapy, 94 (79.6%) with mechanical ventilation, 5 (4.2%) with extracorporeal membrane oxygenation (ECMO), and 23 (19.4%) with renal replacement therapy. Additionally, 112 (94.8%) patients received antibiotics, 71 (60.1%) received antiviral agents such as lopinavir/ritonavir and 92 (77.9%) patients received glucocorticoids (Table 2). The comparisons of treatment for patients in both groups were shown in the Table 2. Compared with control group, patients in treatment group were less likely to receive mechanical ventilation (66.7% vs 84.7%; *p* = 0.028).

Some laboratory markers of inflammation were analyzed during ICU stay. We found that WBC increase and lymphopenia occurred less frequently in the treatment group than control group ((14 [42.4%] vs. 65 [76.4%] and 20 [60.6%] vs. 78 [91.7%]; both *p* < 0.001) (Table 3). Compared with the control group, patients in the treatment group were less likely to have elevated C-reactive protein (CRP) (21 [63.6%] vs. 75 [88.2%], *p* = 0.003). Elevated TNF-a, IL-6, and IL-10 were observed in most patients. Except for TNF-a, elevated IL-6 and IL-10 were less frequently observed in treatment group as compared with control group (19 of 26 [73%] vs. 65 of 68 [95.6%], *p* = 0.004 and 14 of 26 [53.8%] vs. 45 of 66 [80.3%], *p* = 0.018) (Table 3).

## Discussion

This study showed that the mortality rate was significantly lower in patients who received SHG treatment than those who did not. Our results also revealed a lower occurrence rate of organ dysfunction such as ARDS and cardiac injury, lower proportion of patients requiring mechanical ventilation, and shorter time to discharge from ICU in treatment group than control group.

Severe coronavirus disease (COVID-19) is characterized by pulmonary hyper-inflammation and potentially life-threatening systemic inflammatory cytokine storms [22]. Some patients, especially those who are older or have existing chronic medical conditions, may progress rapidly with ARDS, extra-pulmonary organ dysfunction such as acute kidney injury and acute liver injury and need to be admitted to ICU [23,24] . In our cohort, a high occurrence rate of ARDS and other organ dysfunction was observed, indicating that excessive systemically inflammatory response was common in critically ill patients with COVID-19. The mortality rate was higher than that recently reported in other case series and cohort studies [25, 26]. This could reflect the possible lack of critical care resources, especially where facilities are not adequately staffed, given the large number of COVID-19 cases in a short period of time, and the longer follow-up time.

According to current research, a higher pro-inflammatory cytokine storm existed in critical COVID-19 patients inducing excessive systemic inflammatory response, leading to ARDS and other organ dysfunction, and ultimately, death [24-22]. Thus, controlling the local and systemic inflammatory response presumably could reduce the severity and mortality rate of COVID-19 [27, 28]. SHG mainly consists of *Panax ginseng*, which has been shown to have antiviral activity, and *Rheum palmatum* L. stem, which could effectively suppress massive release of inflammatory mediators by inhibition of interactions between spike protein and the angiotensin converting enzyme 2 [29]. Historically, traditional herbal medicines have been effectively used to prevent and control epidemic outbreaks including SARS and H1N1influenza [30, 31]. These findings provide a rationale for clinical application of SHG treatment for severe COVID-19 patients.

In patients with COVID-19, elevated inflammatory cytokines were consistently reported. In our study, we measured the level of some inflammatory cytokines and found that the level of inflammatory cytokines such as TNF-a, IL-6 and IL-10 were significantly increased in most patients. Moreover, the occurrence rates of elevated IL-6 and IL-10 were lower in SHG treatment group as compared to control group. Consistently, the occurrence rate of elevated CRP, which is an inflammatory biomarker, was also lower in treatment group. These results indicated that SHG treatment could decrease the excessive release of inflammatory cytokines and suppress inflammatory response in severe patients with COVID-19. Indeed, *Panax ginseng*, a key component of SHG, was shown to significantly reduce DNA damages induced by oxidative stress in bone marrow cells and peripheral lymphocyte cells by its anti-oxidative stress and anti-inflammatory properties [32]. The SARS-CoV-2 infection activates innate and adaptive immune response. On the other hand, the virus infection might cause deficiency of immune system, as lymphopenia occurred in 35% of non-critical patients and more than 80% of critical patients infected with SARS-CoV-2, respectively [23]. In the current study, lymphopenia occurred in 98 (83%) patients. In addition, lymphopenia was less frequent in treatment group than control group, suggesting that SHG treatment might reverse lymphopenia in severe and critical patients with COVID-19. Moreover, elevated IL-6 and CRP level and lymphopenia were found to be positively associated with disease severity and prognosis of patients with COVID-19 [23, 24, 33]. Indeed, patients in treatment group, who were less likely to have elevated IL-6 and CRP level and lymphopenia, had a lower occurrence rate of organ dysfunction and better prognosis. Our results demonstrated SHG treatment potentially improves outcomes of severe and critical patients with COVID-19 partly through enhancing anti-viral immunity while inhibiting systemic inflammation.

This study has several limitations. First, the present study was a single-center, retrospective, observational study, a type that has a lower standard of evidence and is prone to confounding results and bias. Second, our study did not collect viral load data to determine the efficacy of antiviral effects of SHG treatment. Third, safety data related to the combination treatment with conventional medicine were not evaluated in our study, although these herbs have been used widely in TCM to control infectious diseases. Fourth, due to the retrospective nature of the study, methodologically the two groups had matching and non-matching sample size. Actually, the number of severe patients with COVID-19 dropped dramatically in China after March 2020. Thus, a degree of bias may have been introduced into our two groups. Last, interpretation of our findings might be limited by the sample size. Therefore, future controlled clinical trials are required to validate the findings of our study.

## Conclusion

In summary, our findings suggest that by combination with conventional medicine, SHG treatment might have some beneficial effects such as mortality decrease, reduction in occurrence rate of organ dysfunction, decrease in proportion of patients requiring mechanical ventilation, as well as shorter median length of stay in ICU for severe patients with COVID-19, and warrant further controlled clinical trials to better evaluate the efficacy and safety of SHG treatment for severe COVID-19.

## Figures and Tables

**Table 1:** Demographic, clinical, laboratory and radiographic findings of patients on admission to ICU.

Variable	No. (%)			*p* value

	Total N=118	Treatment group N=33	Control group N=85	
Age, median (IQR), y	68(57-75)	65(56-73)	69(58-75)	0.196
Female gender, No. (%)	39(32.9)	12(36.3)	27(31.7)	0.633
Duration from onset of symptoms to ICU admission, median (IQR), d	10(6-15.2)	13(6.5-17)	10(6-15)	0.571
Comorbidities				
Hypertension	52(44.0)	16(48.4)	36(42.3)	0.679
Coronary heart disease	21(17.7)	3(9.0)	18(21.1)	0.18
Chronic pulmonary disease	9(7.5)	1(3.1)	8(9.3)	0.441
Diabetes	25(21.1)	7(21.2)	18(21.1)	>0.999
Cerebrovascular disease	7(5.7)	4(12.1)	3(3.5)	0.094
Chronic kidney disease	3(2.4)	2(6.0)	1(1.1)	0.188
Liver diseases	0(0)	0(0)	0(0)	>0.999
Malignancy	1(0.8)	0(0)	1(1.1)	>0.999
Smoking	12(10.1)	3(9.0)	9(10.5)	>0.999
Vital signs				
Body temperature, °C(T>37.5°C)	33(27.9)	7(21.2)	26(30.5)	0.366
Heart rate, mean (SD), bpm	94.8(20.4)	90(13.2)	96(22.4)	0.115
Mean blood pressure mean (SD), mmHg	94.5(20.8)	92.9(7.5)	95.1(24.1)	0.62
Respiratory rate, median (IQR), rpm	23(20-27)	22(20-25)	22(20-32)	0.093
Oxygen saturation median (IQR), %	93(86-96)	92.5(87.2-95)	93(85-96)	0.81
Laboratory tests median (IQR)				
White blood cell count, × 10^9^/L	10.5(7.1-14.3)	11.5(7.3-16.0)	9.6(6.6-13.3)	0.144
Lymphocyte count, × 10^9^/L	0.64(0.42-0.96)	0.84(0.42-1.14)	0.58(0.42-0.85)	0.08
Platelet count, ×10^9^/L	167(112-243)	164(95-235)	168(114-248)	0.751
Alanine aminotransferase, U/L	28(18-44)	25(16-45)	28(18-43)	0.701
Aspartate aminotransferase, U/L	34(23-57)	28(19-43)	38(25-67)	0.132
Total bilirubin, mmol/l	11.9(7.8-16.5)	11.1(7.0-16.0)	12.2(8.3-19.2)	0.196
Creatinine, μmol/l	86(64-116)	77(58-128)	87(69-114)	0.262
Blood urea nitrogen, mmol/l	8(5.2-12.6)	6.9(4.1-10.7)	8.4(5.7-13.3)	0.094
Serum sodium (mmol/l)	140(137-144)	141(139-144)	139(136-144)	0.184
Serum potassium (mmol/l)	4.43(4.0-5.0)	4.43(4.1-5.0)	4.48(4.0-4.9)	0.648
Lactate dehydrogenas (U/L)	482(366-673)	492(348-736)	481(366-593)	0.418
Creatine kinase (U/L)	134(74-398)	135(78-419)	132(70-360)	0.421
CK-MB Creatine kinase–MB, U/L	1.7(0.8-2.5)	1.6(0.6-1.9)	1.8(1.1-7.1)	0.204
Fibrinogen, g/l	4.7(3.2-5.9)	4.4(3.0-5.2)	4.7(3.2-6.1)	0.274
Prothrombin time, s	15.6(14.5-17.8)	16.1(14.6-18.3)	15.5(14.5-17.4)	0.251
Activated partial thromboplastin time, s	40.7(35.7-45.7)	40(35.6-46.1)	41(35.9-45.6)	0.97
D-dimmer, mg/l	3.4(1.4-12.6)	4.6(1.6-8.1)	3.2(1.3-14.8)	0.996
C-reactive protein, mg/l	87(37-144)	67(12-134)	97(41-148)	0.117
Procalcitonin, ng/ml	0.2(0.1-1.1)	0.2(0.1-2.2)	0.2(0.1-0.87)	0.559
Imaging features				
Consolidation	48(40.6)	14(42.4)	34(40.0)	0.836
Ground-glass opacity	42(35.5)	10(30.3)	32(37.6)	0.524
Bilateral pulmonary infiltration	93(78.7)	25(75.7)	68(80.0))	0.622

Abbreviations: ICU, intensive care unit, IQR, interquartile range.

Chronic pulmonary disease was defined as chronic obstructive pulmonary disease, asthma, or chronic bronchitis.

**Table 2:** Complications, treatments and outcomes of patients.

	No. (%)			*p* value

	Total N=118	Treatment group N=33	Control group N=85	
Complications				
ARDS	66(55.8)	12(36.3)	54(63.5)	**0.012**
Liver dysfunction	18(14.2)	4(12.1)	14(16.4)	0.555
Acute kidney injury	36(30.5)	8(24.2)	28(32.9)	0.357
Cardiac injury	37(31.3)	5(15.1)	32(37.6)	**0.026**
Coagulopathy	29(24.5)	9(27.2)	20(23.5)	0.812
Shock	27(22.8)	5(15.1)	22(25.8)	0.328
Treatments				
High-flow nasal cannula oxygen therapy	20(16.9)	9(27.2)	11(12.9)	0.098
Mechanical ventilation	94(79.6)	22(66.7)	72(84.7)	**0.028**
Non-invasive	23(19.3)	4(12.1)	19(22.3)	0.301
Invasive	71(60.1)	18(54.5)	53(62.3)	0.436
ECMO	5(4.2)	2(6.0)	3(3.5)	0.618
Antiviral therapy	71(60.1)	21(63.6)	50(58.8)	0.679
Antibiotics	112(94.8)	31(93.9)	82(95.2)	0.671
Glucocorticoid therapy	92(77.9)	24(72.7)	68(80)	0.459
Renal replacement therapy	23(19.4)	7(21.2)	16(18.8)	0.798
Outcomes				
Discharged from ICU	29(24.5)	14(42.4)	15(17.6)	**0.008**
Died in ICU	83(70.3)	15(45.4)	68(80.0)	**0.0002**
Still in ICU as of 4/22/2020^a^	6(5.2)	4(12.2)	2(2.4)	0.051
Length of ICU stay at study end point^b^				
Died, median (IQR) (d)	9(6-17)	18(12-30)	9(6-13)	**0.0002**
Discharged alive, median (IQR), (d)	67(27-76)	32(20-73)	76(63-79)	**0.0074**

Abbreviations: ICU, intensive care unit; ARDS, acute respiratory distress syndrome;

ECMO, extracorporealmembrane oxygenation; IQR, interquartile range.

^a^Patients were admitted between 1/28/2020 and 3/28/2020, with follow-up through 4/22/2020.

^b^Length of stay begins with admission time and ends with discharge time, time at death, or on the last day of data collection for the study.

Boldface values were considered statistically significant.

**Table 3:** Inflammatory markers in patients during ICU stay.

	No. / total No. (%)^a^			*p* value

	Total N=118	Treatment group N=33	Control group N=85	
White blood cell count, × 109/L				
<3.5	11(9.2)	3(9.0)	8(9.4)	>0.999
>9.5	79(66.8)	14(42.4)	65(76.4)	**0.0009**
Lymphocyte count, × 109/L				
<1.1	98(83.0)	20(60.6)	78(91.7)	**0.0002**
>3.2	1(0.8)	0(0)	1(1.1)	>0.999
Procalcitonin, ng/ml				
increased	53(44.8)	14(42.4)	39(45.8)	0.837
C-reactive protein, mg/l				
increased	96(81.3)	21(63.6)	75(88.2)	**0.003**
TNF-a, pg/ml				
increased	67/93(71.9)	22/26(84.6)	45/67(67.1)	0.123
IL-6, pg/ml				
increased	87/94(92.5)	19/26(73.0)	65/68(95.6)	**0.004**
IL-10, pg/ml				
increased	59/82(72.8)	14/26(53.8)	45/66(80.3)	**0.018**

Abbreviations: ICU, intensive care unit.

^a^For some variables, not available for all patients.

Boldface values were considered statistically significant.
